# Self-report and proxy reports in survey data on female genital mutilation, Senegal

**DOI:** 10.2471/BLT.24.292383

**Published:** 2025-04-08

**Authors:** Kathrin Weny, Romesh Silva, Stefanie J Klug

**Affiliations:** a Chair of Epidemiology, TUM School of Medicine and Health, Technical University of Munich, Georg-Brauchle-Ring, Munich 80992, Germany.; b United Nations Population Fund, New York, United States of America.

## Abstract

**Objective:**

To assess the quality and consistency of reported age patterns of female genital mutilation in self- and proxy-reported survey data.

**Methods:**

We used 10 Demographic and Health Surveys (DHS) from 2005 to 2023 in Senegal. These surveys contained information on female genital mutilation status and age at experiencing this practice for women who reported data on themselves and daughters for whom data were reported by their mothers. We assessed data quality by completeness of information on age at female genital mutilation in a logistic regression analysis. We compared the occurrence of age heaping across DHS and individual survey characteristics such as education, age cohort and completeness of date of birth reporting. We estimated the median age at female genital mutilation of daughters and women to assess the consequences of differences in data quality for the interpretation of survey data on this practice.

**Findings:**

Self-reported data were more prone to incomplete reporting of age at female genital mutilation and age heaping than proxy-reported data. These findings held true across individual survey characteristics and different DHS. The estimates for median age at female genital mutilation were susceptible to differences in data quality of age at female genital mutilation of daughters and women.

**Conclusion:**

Self-reported data on age at female genital mutilation are of lower quality than proxy-reported data. These differences potentially distort trend estimates of age at female genital mutilation. Caution is needed when combining self- and proxy-reported survey data on female genital mutilation.

## Résumé

**Objectif:**

Évaluer la qualité et la cohérence des schémas de mutilations génitales féminines par âge dans les données d’enquête autodéclarées et déclarées par un tiers.

**Méthodes:**

Nous nous sommes basés sur 10 enquêtes démographiques et sanitaires (EDS) réalisées entre 2005 et 2023 au Sénégal. Ces enquêtes contenaient des informations sur l’état des mutilations génitales féminines et l’âge auquel elles ont été pratiquées pour les femmes qui ont déclaré des données les concernant et pour les filles dont les données ont été déclarées par leur mère. Nous avons évalué la qualité des données en fonction de l’exhaustivité des informations sur l’âge au moment des mutilations génitales féminines dans le cadre d’une analyse de régression logistique. Nous avons comparé l’occurrence de l’arrondissement des âges selon plusieurs EDS et selon des caractéristiques d’enquêtes individuelles, telles que l’éducation, la cohorte d’âge et l’exhaustivité de la déclaration de la date de naissance. Nous avons estimé l’âge médian des filles et des femmes au moment des mutilations génitales féminines afin d’évaluer les conséquences des différences de qualité des données sur l’interprétation des données d’enquête relatives à cette pratique.

**Résultats:**

Les données autodéclarées étaient davantage susceptibles d’être incomplètes que les données déclarées par un tiers en ce qui concerne l’âge au moment des mutilations génitales féminines et l’arrondissement des âges. Ces résultats s’appliquent à toutes les caractéristiques d’enquêtes individuelles et à différentes EDS. Les estimations de l’âge médian au moment des mutilations sexuelles féminines étaient sensibles aux différences de qualité des données pour les filles comme pour les femmes.

**Conclusion:**

Les données autodéclarées sur l’âge au moment des mutilations sexuelles féminines sont de moins bonne qualité que les données déclarées par un tiers. Ces différences sont susceptibles de fausser les estimations des tendances de l’âge des mutilations sexuelles féminines. Il convient d’être prudent lorsque l’on combine des données d’enquête autodéclarées et celles déclarées par un tiers en matière de mutilations sexuelles féminines.

## Resumen

**Objetivo:**

Evaluar la calidad y la coherencia de los datos sobre los patrones de edad en la mutilación genital femenina, según se trate de autodeclaraciones o de declaraciones por terceros en encuestas.

**Métodos:**

Se analizaron diez encuestas demográficas y de salud (DHS) realizadas en Senegal entre 2005 y 2023. Estas encuestas contenían información sobre el estado de mutilación genital femenina y la edad en la que se había experimentado esta práctica, tanto en mujeres que aportaron datos sobre sí mismas como en hijas cuyos datos fueron proporcionados por sus madres. Se evaluó la calidad de los datos mediante un análisis de regresión logística que consideró la integridad de la información sobre la edad en el momento de la mutilación. Asimismo, se comparó la concentración de respuestas en edades redondas entre las distintas DHS y en función de características individuales como el nivel educativo, la cohorte de edad y la precisión en la notificación de la fecha de nacimiento. Se estimó la edad mediana al momento de la mutilación genital femenina en mujeres y en hijas, con el fin de evaluar las consecuencias de las diferencias en la calidad de los datos sobre la interpretación de esta práctica en las encuestas.

**Resultados:**

Las autodeclaraciones presentaron una mayor propensión a la falta de información sobre la edad en el momento de la mutilación genital femenina y a la concentración de respuestas en edades redondas, en comparación con las declaraciones por terceros. Estas diferencias se mantuvieron constantes entre distintas características individuales y entre las diversas DHS. Las estimaciones de la edad mediana al momento de la mutilación resultaron sensibles a las variaciones en la calidad de los datos reportados sobre mujeres y sobre hijas.

**Conclusión:**

Las autodeclaraciones sobre la edad en el momento de la mutilación genital femenina presentan una calidad inferior respecto a las declaraciones por terceros. Estas diferencias pueden distorsionar las estimaciones de tendencias sobre la edad en que ocurre esta práctica. Se recomienda cautela al combinar datos autodeclarados y datos declarados por terceros en las encuestas sobre mutilación genital femenina.

## ملخص

الغرض تقييم جودة وتناسق أنماط الأعمار المُبلّغ عنها لختان الإناث في بيانات المسوح الذاتية والتقارير بالوكالة.

الطرق قمنا بالاستعانة بعشرة مسوح سكانية وصحية (DHS) في الفترة من 2005 إلى 2023، في السنغال. تضمنت هذه المسوحات معلومات عن حالة ختان الإناث، وعمر التعرض لهذه الممارسة لدى السيدات اللواتي أبلغن عن بياناتهن الخاصة، وكذلك عن بناتهن اللواتي أبلغت أمهاتهن عن بياناتهن. قمنا بتقييم جودة البيانات من خلال اكتمال المعلومات المتعلقة بالعمر عند إجراء ختان الإناث باستخدام تحليل الانحدار اللوجستي. كما قمنا بمقارنة حدوث تراكم الأعمار عبر المسوح السكانية والصحية، وخصائص المسوح الفردية مثل التعليم، والفئة العمرية، واكتمال الإبلاغ عن تاريخ الميلاد. وقدّرنا متوسط العمر عند إجراء ختان الإناث للبنات والنساء، وذلك لتقييم آثار الاختلاف في جودة البيانات على تفسير بيانات المسوح حول هذه الممارسة.

النتائج كانت البيانات المُبلّغ عنها ذاتيًا أكثر عرضةً للإبلاغ غير الكامل عن العمر عند ختان الإناث وتراكمه، مقارنةً بالبيانات المُبلّغ عنها بالوكالة. وقد ثبتت صحة هذه النتائج عبر خصائص المسح الفردي وبيانات المسوح السكانية والصحية المختلفة. وكانت تقديرات متوسط العمر عند إجراء ختان الإناث، عُرضة للاختلافات في جودة بيانات العمر عند ختان الإناث للبنات والسيدات.

الاستنتاج تُعدّ البيانات المُبلّغ عنها ذاتيًا عن العمر عند إجراء ختان الإناث أقل جودةً من البيانات المُبلّغ عنها بالوكالة. وقد تُشوّه هذه الاختلافات تقديرات اتجاهات العمر عند ختان الإناث. لذا، يجب توخي الحذر عند الجمع بين بيانات المسح المُبلّغ عنها ذاتيًا، والبيانات المُبلّغ عنها بالوكالة، حول ختان الإناث..

## 摘要

**目的:**

评估自述报告和代述报告调查数据中关于残割女性生殖器的年龄分布相关报告的质量和一致性。

**方法:**

我们运用了自 2005 年至 2023 年期间在塞内加尔开展的 10 次人口和保健调查 (DHS) 相关数据。此类调查数据包含了在接受残割女性生殖器手术时女性（自行汇报数据的妇女以及由其母亲代为汇报数据的女儿）的身份和年龄相关信息。在逻辑回归分析中，我们按照接受残割女性生殖器手术时的女性年龄相关信息的完整性对数据质量进行了评估。我们对 DHS 和个体特征调查（例如受教育程度、年龄组群以及出生日期报告完整性）中出现的年龄堆积现象进行了对比。我们估算了在接受残割女性生殖器手术时妇女及其女儿的中位年龄，以评估数据质量差异对解释该手术相关调查数据的影响。

**结果:**

与代述报告数据相比，自述报告数据更容易出现与残割女性生殖器有关的年龄报告不完整和年龄堆积现象。此类研究结果适用于个体特征调查和各种 DHS。在估算接受残割女性生殖器手术时妇女及其女儿的中位年龄时，易受年龄相关数据质量差异的影响。

**结论:**

与代述报告数据相比，关于接受残割女性生殖器手术时女性年龄的自述报告数据的质量更低。此类差异可能会导致接受残割女性生殖器手术时的女性年龄趋势估算失真。在汇总有关残割女性生殖器的自述报告和代述报告调查数据时需保持谨慎。

## Резюме

**Цель:**

Оценить качество и однородность возрастных паттернов, прослеживаемых в данных опросов о калечащих операциях на женских половых органах (по информации, предоставленной непосредственно женщинами, которые подверглись данной процедуре, и третьими лицами).

**Методы:**

Были использованы данные 10 опросов, посвященных демографическим и медико-санитарным обследованиям (Demographic and Health Surveys, DHS), которые были проведены в Сенегале в период с 2005 по 2023 год. Опросы содержали информацию о факте калечащей операции на женских половых органах и возрасте, в котором была проведена данная процедура. Сведения предоставлялись непосредственно женщинами, которые подверглись такой операции, либо матерями, если операция проводилась их дочерям. Авторы провели логистический регрессионный анализ для оценки качества данных по полноте сведений о возрасте, в котором проводилась калечащая операция на женских половых органах. Сравнивалась частота одинаковых сообщений о возрасте вмешательства, согласно данным DHS, и индивидуальные характеристики респонденток, такие как уровень образования, возрастная когорта и полнота сведений о дате рождения. Для оценки воздействия различного качества данных на интерпретацию этих опросов в отношении указанной практики было рассчитано медианное значение возраста проведения калечащих операций на женских половых органах для женщин и их дочерей.

**Результаты:**

Данные, сообщаемые самими женщинами, подвергшимися калечащим операциям на половых органах, чаще оказывались неполными в части сообщения о возрасте проведения процедуры, а также чаще указывался один и тот же возраст, в то время как данные, сообщаемые третьими лицами, носили более полный характер. Обнаруженные закономерности подтверждались при анализе индивидуальных характеристик участниц и различных опросов DHS. Выявленное медианное значение возраста проведения калечащих операций на женских половых органах зависело от различий в качестве данных о возрасте проведения данной процедуры, которые предоставлялись женщинами о себе и матерями о своих дочерях.

**Вывод:**

Данные о возрасте проведения калечащих операций на женских половых органах, предоставленные самими женщинами, подвергшимися этой процедуре, отличаются меньшей достоверностью по сравнению с данными, которые матери сообщают относительно своих дочерей. Эти различия могут искажать тенденции оценки возраста проведения калечащих операций на женских половых органах. При совместном анализе данных о калечащих операциях на женских половых органах, полученных от самих женщин и третьих лиц, следует проявлять осторожность.

## Introduction

Female genital mutilation is internationally defined as “all procedures that involve partial or total removal of the external female genitalia, or other injury to the female genital organs for non-medical reasons”.^1^^–^^3^ This practice can have severe health consequences, such as bleeding, infections and risks during birth for both women and their children.^1^ Beyond its medical implications, female genital mutilation is recognized as a violation of women’s and girls’ human rights, namely their right to health, security and physical integrity.^1^^,^^3^

In 2008, 10 United Nations (UN) agencies called for an end to the practice^4^ and the United Nations Population Fund (UNFPA) and United Nations Children’s Fund (UNICEF) launched the Joint Programme on the Elimination of Female Genital Mutilation.^5^ In 2015, Member States of the UN pledged to eliminate the practice by 2030.^6^ Progress towards elimination is measured with data from Demographic and Health Surveys (DHS), Multiple Indicator Cluster Surveys (MICS) and national surveys which collect data on female genital mutilation in 31 countries.^7^^–^^9^

**Fig. 1:**
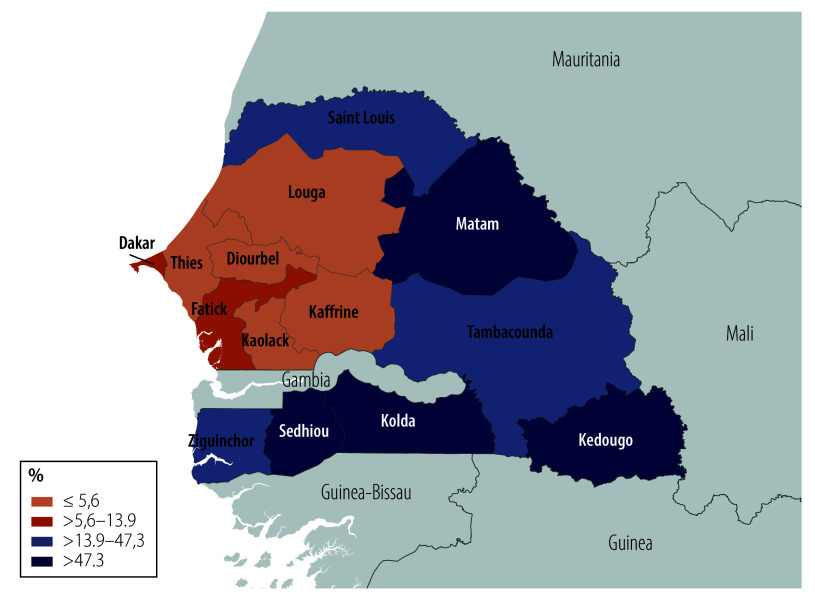
Percentage of women aged 15–49 years who experienced female genital mutilation, by region, Senegal, 2023

Source: Senegal Demographic and Health Survey 2023.^15^

Female genital mutilation is documented in various countries, such as among migrant communities in Europe^10^ and the United States of America,^11^ as well as in Colombia, India, Malaysia, Oman, Saudi Arabia and the United Arab Emirates.^7^ Estimated prevalence rates among women aged 15–49 years range from 0.3% in Uganda^12^ up to 98% and 99% in Somalia’s north-east zone and Somaliland, respectively.^13^^,^^14^ According to the 2023 Demographic and Health Survey in Senegal, about one in five women aged 15–49 years has experienced female genital mutilation.^15^ This proportion is a decline after almost two decades of stagnant numbers on female genital mutilation when roughly one in four women was subjected to the practice.^16^ Female genital mutilation is not evenly spread across the country. In Diourbel, for example, less than 1% of women are estimated to have experienced female genital mutilation, while in Matam, 83% are affected (Fig. 1).^15^

UNFPA maintains that the age at which women and girls experience female genital mutilation is important for context-specific and effective interventions. Where female genital mutilation is practised on adolescents, programmes can include them directly. On the other hand, where girls are very young, interventions may rather target members of their community or parents and be linked to ante- and perinatal health programmes.^17^ The age at which female genital mutilation occurs depends on the context: it may be at birth (e.g. in Mauritania), but girls remain at risk up to late adolescence (e.g. in Kenya).^2^^,^^18^^,^^19^ In Senegal, female genital mutilation mostly occurs early, before 5 years of age.^2^^,^^18^^,^^19^

The DHS and MICS include women aged 15–49 years to assess their experience of female genital mutilation.^20^ Since 2010, questions to women about all their living daughters younger than 15 years were also systematically included.^18^^,^^21^ This inclusion allows the evolution of female genital mutilation in recent years to be estimated,^18^^,^^21^ if appropriate statistical techniques are used.^2^^,^^22^

Since data on daughters younger than 15 years are gathered from their mothers (proxy-reported), while data on women are reported by themselves (self-reported),^23^ these different collection methods may influence results, for example through differences in the accuracy of recall.^22^ Women, especially older women, may not remember the context and consequences of their own experience of female genital mutilation in detail.^20^ This issue can be particularly problematic when women are asked about specifics of their experience of female genital mutilation.^20^

While we expect these differences in data quality, their extent and therefore implications for the use of data on female genital mutilation are not obvious without a thorough analysis. Given the importance of data on the age at experiencing female genital mutilation as outlined earlier, this information is crucial for the interpretation of survey data on female genital mutilation. By comparing proxy-reported data on daughters to self-reported data on women, we aimed to provide answers on the quality and interpretation of survey data of female genital mutilation. This information will help to clarify the validity of estimates of changes in the incidence risk of female genital mutilation over time.

## Methods

This secondary data analysis used population-level household survey data from 10 DHS in Senegal between 2005 and 2023. We chose Senegal because of its unique data ecosystem. Usually, DHS and MICS are collected in about 5-year intervals. Senegal, however, conducted a continuous DHS with yearly surveys between 2011 and 2019.^24^ Our analysis included the two DHS female genital mutilation modules, as well as sampling design variables and individual survey characteristics of women and daughters from the standard DHS woman’s questionnaire. In the women’s female genital mutilation module, women aged 15–49 years are asked about their experience of female genital mutilation, among others: if and when they experienced female genital mutilation; who performed the procedure; and which type of female genital mutilation was done on them. In the daughters’ module, mothers are asked about the female genital mutilation experience of all their living daughters younger than 15 years. Not all DHS female genital mutilation modules in Senegal have the same format. The 2005 DHS did not include data on daughters,^25^ while the 2012–2013 DHS did not collect data on women.^26^

Similar to a previous study,^27^ we combined all 10 DHS to construct a pooled data set by denormalizing the sample weights provided with each DHS. We adjusted for the likelihood of an observation being included in the survey. We made this adjustment based on the total number of women aged 15–49 years and girls aged 0–14 years in Senegal in the year the survey was conducted, as all of them were eligible for one of the two female genital mutilation modules. We retrieved the population data necessary for denormalization from the UN’s Population Division. As DHS are standardized across time, our data harmonization largely focused on adjusting variable names to create the pooled data set. We used the same approach employed in previous studies.^2^^,^^3^

In the women’s module, women may give an approximate answer for their age at experiencing female genital mutilation, usually during infancy. We did not eliminate these responses from the data set but redistributed them based on the distribution of precise responses by women on their age at experiencing female genital mutilation between the ages of 0 and 5 years for each DHS in this analysis (see previous studies and online repository for more details).^2^^,^^3^^,^^28^ We excluded cases with inconsistent female genital mutilation reporting, that is, where age at experiencing female genital mutilation was older than the current age of the women or daughter.

To compare data quality of the women’s and daughters’ modules, we first assessed completeness of age at experiencing female genital mutilation. Completeness was calculated as the percentage of observations for which the age at experiencing female genital mutilation was reported. We analysed the data by age of women and daughters and survey module.

To understand what factors significantly affect completeness of reporting of age at experiencing female genital mutilation by women and by mothers for their daughters, we used logistic regression analysis. We controlled for the specific DHS, survey module, age of women and daughters, educational status of women, and the completeness of date of birth reporting by women for themselves. Completeness of date of birth reporting is a DHS indicator that records if the date of birth of the survey respondent had to be imputed by the DHS due to inconsistencies or missing data during data collection. In our analysis, we distinguished between observations for which the date of birth had to be imputed and for which the date of birth was completely reported in the survey.

We then analysed age heaping in age at experiencing female genital mutilation. Age heaping is an indicator of data quality and is defined as the extent to which the recording of age in a data set indicates disproportionate digit preference for certain ages, and suggests that the exact age is probably unknown.^29^ We calculated the magnitude of age heaping by comparing the number of cases of female genital mutilations reported at 5, 10 and 15 years of age with the number that was expected under smooth transition from adjacent ages, that is, from ages 4 and 6 years in the case of age at female genital mutilation of 5 years. We analysed age heaping by DHS, survey module and individual characteristics, namely, education of women, completeness of date of birth reporting of women, and birth cohort of women and daughters.

We then calculated median age at experiencing female genital mutilation for women and daughters by birth cohort to compare their respective patterns of age at female genital mutilation.

We used an R survey package (R Foundation, Vienna, Austria)^30^ for statistical assessment to account for the complex sampling design of DHS.

## Results

This analysis includes 10 DHS with a sample size of 190 053: 107 924 women were responding for themselves, 34% (37 170) of whom reported having experienced female genital mutilation, and 82 129 observations were reported by mothers for their daughters, 20% (16 660) of whom had experienced female genital mutilation (Table 1). Overall, 62% (118 081/190 053) of the women and mothers surveyed reported no education and 64% (122 212/190 053) of date of birth reports were incomplete (Table 2). The oldest birth cohort included in the analysis was 1960–1969 and the youngest was born after 2020 (Table 3). Less than 1% (872/190 053) of birth cohort information was missing (Table 3). Overall, 18% (6847/37 170) of women and 47% (7858/16 660) of daughters experienced female genital mutilation in the first year of life. Data were missing on age at female genital mutilation for 6% (2356/37 170) of women and 1% (239/16 660) of daughters (Table 4).

**Table 1:** Frequency of female genital mutilation by DHS year and module, Senegal, 2005–2023

DHS year	Women’s module			Daughters’ module			Both modules	
	All observations, unweighted	Experienced female genital mutilation, no. (%)		All observations, unweighted	Experienced female genital mutilation, no. (%)		All observations, unweighted	Experienced female genital mutilation, no. (%)
2005^25^	14 599	5 274 (36)		NA	NA		14 599	5 274 (36)
2010–2011^31^	15 688	5 689 (36)		10 499	1 971 (19)		26 187	7 660 (29)
2012–2013^26^	NA	NA		2 805	622 (22)		2 805	622 (22)
2014^32^	8 488	2 858 (34)		7 879	1 454 (18)		16 367	4 312 (26)
2015^33^	8 851	3 155 (36)		8 200	1 787 (22)		17 051	4 942 (29)
2016^34^	8 865	3 128 (35)		7 916	1 618 (20)		16 781	4 746 (28)
2017^35^	16 787	5 719 (34)		15 028	3 065 (20)		31 815	8 784 (28)
2018^36^	9 414	3 094 (33)		8 379	1 759 (21)		17 793	4,853 (27)
2019^37^	8 649	3 303 (38)		7 828	1 878 (24)		16 477	5 181 (31)
2023^15^	16 583	4 950 (30)		13 595	2 506 (18)		30 178	7 456 (25)
Total	107 924	37 170 (34)		82 129	16 660 (20)		190 053	53 830 (28)

DHS: Demographic and Health Surveys; NA: not applicable.

Note: We have marked modules not done in a certain year as NA. These data exclude seven inconsistent observations for which the reported age at experiencing female genital mutilation was older than the age at the time of the survey.

**Table 2:** Observations by women’s education and completeness of date of birth reporting, Senegal, 2005–2023

Variable	No. (%) of observations, unweighted (*n* = 190 053)
**Highest education of women**	
None	118 081 (62)
Primary	37 849 (20)
Secondary	31 322 (16)
Higher	2795 (1)
Other	3 (0)
Missing	3 (0)
**Completeness of date of birth reporting for themselves**	
Complete	67 841 (36)
Computed	122 212 (64)

Sources: Senegal DHS 2005,^25^ 2010–2011,^31^ 2012–2013,^26^ 2014,^32^ 2015,^33^ 2016,^34^ 2017,^35^ 2018,^36^ 2019,^37^ 2023.^15^

**Table 3:** Number of observations (unweighted) for women and daughters, by birth cohort, Senegal, 2005–2023

Birth cohort year	Women	Daughters	% of total
1960–1969	6 431	0	3
1970–1979	20 022	0	11
1980–1989	34 192	0	18
1990–1999	33 477	421	18
2000–2009	12 930	37 265	26
2010–2019	0	41 174	22
After 2020	0	3 269	2
Missing	872	0	< 1
**Total**	**107 924**	**82 129**	**100**

Sources: Senegal DHS 2005,^25^ 2010–2011,^31^ 2012–2013,^26^ 2014,^32^ 2015,^33^ 2016,^34^ 2017,^35^ 2018,^36^ 2019,^37^ 2023.^15^

**Table 4:** Number of observations by age at experiencing female genital mutilation, Senegal, 2005–2023

Age at female genital mutilation, years	No. (%)	
	Women	Daughters
< 1	6847 (18)	7 858 (47)
1–5	22 852 (61)	7 993 (48)
6–10	4 205 (11)	556 (3)
11–15	817 (2)	14 (0)
> 15	93 (0)	0 (0)
Missing	2 356 (6)	239 (1)
**Total**	**37 170 (100**)	**16 660** (**100**)

Sources: Senegal DHS 2005,^25^ 2010–2011,^31^ 2012–2013,^26^ 2014,^32^ 2015,^33^ 2016,^34^ 2017,^35^ 2018,^36^ 2019,^37^ 2023.^15^

### Completeness of data

**Fig. 2:**
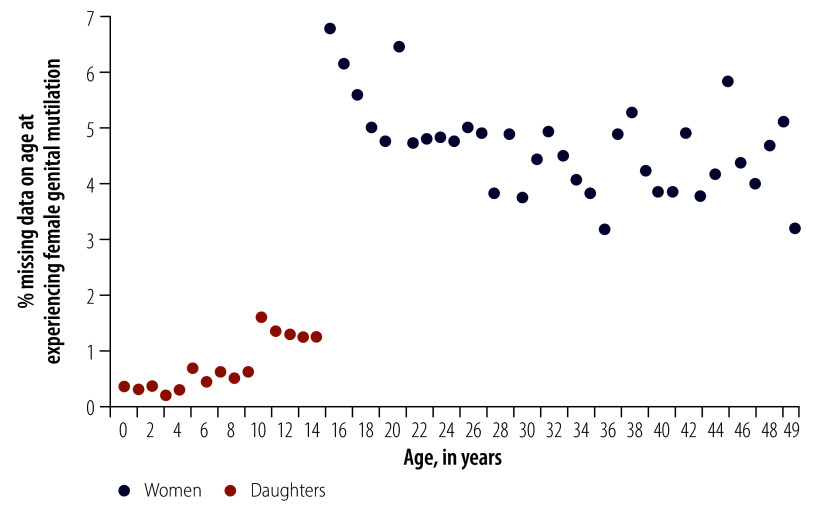
Proportion of observations with missing data on age at experiencing female genital mutilation, by survey module and age, Senegal

Note: This figure builds on an earlier data visualization in work by some of the authors.^17^

Within the daughters’ module, the percentage of missing data increased with the age of daughters. Yet, women showed a higher prevalence of missing data in age at experiencing female genital mutilation than daughters at all ages (Fig. 2). A discontinuous jump between ages 14 and 15 years, that is between the women’s and daughters’ module, is discernible.

The 2014, 2017, 2018, 2019 and 2023 DHS as well as the women’s module were significantly associated with the completeness of reporting age at experiencing female genital mutilation (Table 5). The odds of complete reporting of age at female genital mutilation were lower in the 2014 and 2023 DHS than the 2005 DHS, while the odds were greater in the 2017, 2018 and 2019 DHS. The odds of complete reporting of age at female genital mutilation were also significantly lower in the women’s module compared with the daughters**’** module (Table 5).

**Table 5:** Association between individual and survey characteristics and completeness of reporting age at experiencing female genital mutilation, Senegal, 2005–2023

Characteristic	OR (95% CI)
**Education**	
No education (reference)	1.00
Primary	1.00 (0.82–1.21)
Secondary	1.06 (0.88–1.27)
Higher	0.76 (0.48–1.20)
**Survey year**	
2005 (reference)	1.00
2010–2011	0.87 (0.59–1.28)
2012–2013	1.73 (0.42–7.10)
2014	0.62 (0.39–0.99)
2015	0.91 (0.56–1.49)
2016	1.04 (0.66–1.63)
2017	2.22 (1.51–3.26)
2018	2.07 (1.34–3.20)
2019	4.25 (2.57–7.01)
2023	0.35 (0.25–0.51)
**Module**	
Daughters’ (reference)	1.00
Women’s	0.16 (0.11–0.22)
**Age of women and daughters, in years**	1.00 (1.00–1.01)
**Date of birth**	
Imputed (reference)	1.00
Completely reported in survey	0.86 (0.72–1.03)

CI: confidence interval; OR: odds ratio.

### Age heaping

**Fig. 3:**
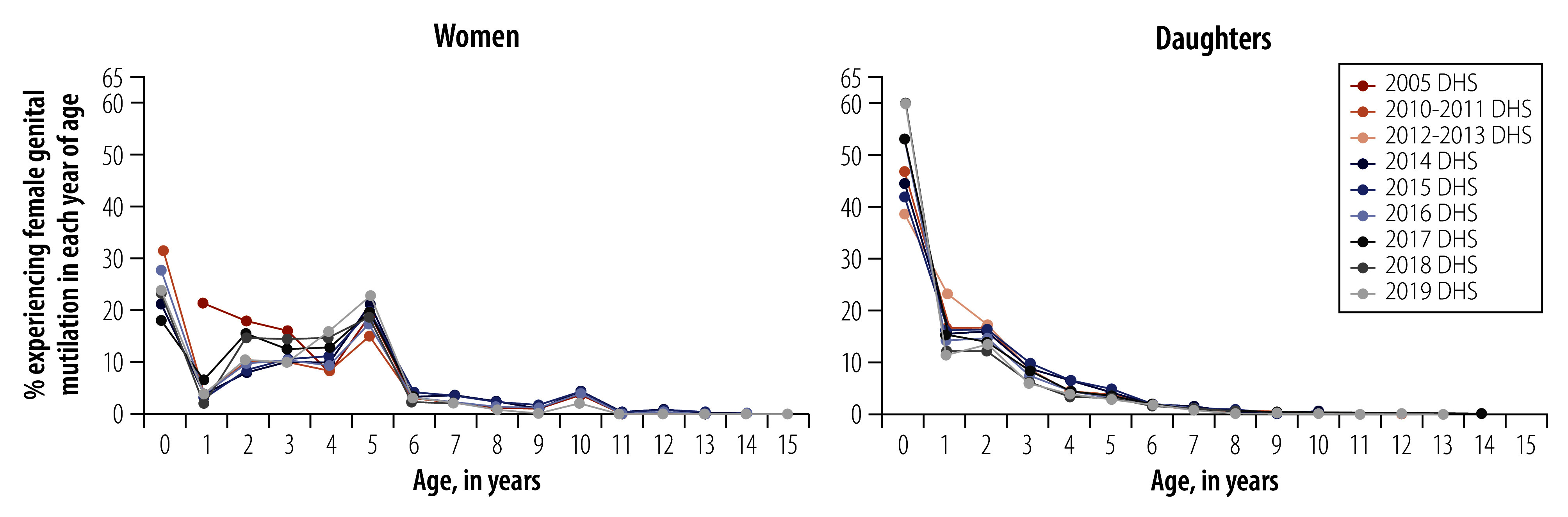
Age structure of female genital mutilation for women and daughters, by survey year, Senegal, 2005–2019

For women’s self-reported data, the occurrence of female genital mutilation at age 5 years is roughly 2.5 times more likely than expected under smooth distribution of age at female genital mutilation, while the occurrence of age 10 years is almost four times more likely and of age 15 years almost three times more likely. For daughters, age 5 years is reported slightly more often than expected, while age 10 years is almost twice as likely to be reported (Fig. 3).

DHS: Demographic and Health Survey.

Note: This age structure excludes 77 observations with an age at experiencing female genital mutilation older than 15 years. The 2005 DHS did not include data on daughters, while the 2012–2013 DHS did not collect data on women.

Age heaping was also analysed by survey module and individual characteristics, such as education of women (online repository),^28^ completeness of date of birth reporting by women for themselves (online repository)^28^ and birth cohort of women and daughters (online repository).^28^ Except for survey module, none of these variables determined the extent of age heaping and the pattern of age at experiencing female genital mutilation.

### Median age

We calculated the median age at female genital mutilation for mothers and daughters by their respective birth cohorts. Across both modules, median age at experiencing female genital mutilation of all observations was close to the median for women for the cohorts from 1960–1969 to 1990–1999. Thereafter, the median age was determined from the daughters’ module. Thus, median age at female genital mutilation decreased from 3 or 4 years for women born between 1960 and 1969 to 1 year or younger for daughters born from 2010 to 2019 and after 2020. However, when calculating median age at female genital mutilation by survey module, the median age at female genital mutilation was fairly constant across cohorts of women and daughters observed in our study (Table 6).

**Table 6:** Median age at experiencing female genital mutilation by module and birth cohort, Senegal

Birth cohort	Median age, years		
	Both modules	Women’s module	Daughters’ module
1960–1969	3	3	NA
1970–1979	4	4	NA
1980–1989	3	3	NA
1990–1999	3	3	1
2000–2009	1	3	1
2010–2019	0	NA	0
After 2020	1	NA	1

NA: not applicable.

## Discussion

When comparing two types of data from successive surveys, it is challenging to determine which is closer to the true value.^29^ In the case of female genital mutilation, clinical examination can determine the true status. In the past, clinical examination found varying degrees of inconsistency between reported and actual female genital mutilation status.^38^ Studies in some contexts found significant inconsistencies between reported and observed female genital mutilation status,^39^ while others found remarkable concordance.^40^ In the case of age at experiencing female genital mutilation, such a comparison is not possible, as this age cannot be observed retrospectively. However, reliable estimates of the ages at which girls and adolescents are at risk of female genital mutilation is important because it is a crucial variable to design effective, context-specific programmes against female genital mutilation.^17^

To address this problem, we compared data quality of reported age at experiencing female genital mutilation. We followed previous data quality assessments of DHS data that used completeness and age heaping as indicators.^29^ While completeness indicates the share of observations for which age at female genital mutilation is reported, we calculated age heaping as the deviation of the expected number of female genital mutilation cases at 5, 10 and 15 years of age, under smooth transition between adjacent ages. We chose this method as age heaping indices such as the Whipple or Myers index cannot be applied to age ranges younger than 10 years.^41^ Hence, a comparison to age heaping in the context of age reporting is not possible. However, the higher number of cases for which age at experiencing female genital mutilation is reported at 5, 10 or 15 years in the women’s module compared with the daughters’ module hints at more approximate answers by women with respect to their own age when they experienced female genital mutilation compared with the age the practice occurred for their daughters.^29^

We performed our analysis by DHS to discern if inconsistencies came from differences in successive surveys, for example through increased data quality of DHS over time. Our analysis, however, demonstrated remarkable consistency across DHS from 2005 to 2023, suggesting reliability of DHS data.

The age of women at the time of the survey may lead to inconsistent results as older age increases the time passed since female genital mutilation occurred.^38^ In our analysis, the percentage of women who gave no answer on their age at experiencing female genital mutilation was relatively constant across age. Adolescent women did not have a lower probability of reporting their age at female genital mutilation as missing compared with women in their late 40s. Furthermore, analysis of the effect of birth cohort on reporting quality showed that birth cohort could be excluded as a source of inconsistencies. In contrast, data on age at female genital mutilation from the women’s module was much more likely to be missing than data from the daughters’ module.

Education has been cited as an important factor in the quality of age reporting.^42^ However, our analysis does not support an effect of education on the quality of reporting. Women with higher educational status were neither more likely to report their age at experiencing female genital mutilation, nor more likely to avoid age heaping.

Similarly, missing age at female genital mutilation and age heaping were not correlated with the completeness of date of birth reported by women. This finding indicates that overall reporting quality measured by completeness of date of birth reporting was not directly linked to the quality of reporting on female genital mutilation.

We conclude therefore that across all variables, data on age at female genital mutilation for daughters provided by their mothers were more reliable than the women’s data as reported for themselves. As female genital mutilation mostly occurs in early childhood in Senegal, this result is not surprising and is in line with previous assumptions.^20^^,^^22^^,^^38^ Data on daughters seemed easier to recall for mothers compared with data on experiences in their own childhood.

These differences in data quality affect the interpretation of data on age at female genital mutilation. By further analysing the median age at experiencing female genital mutilation for both modules separately, we found higher age estimates for women compared with daughters. Notably, we distributed approximate answers of women on their age at experiencing female genital mutilation among the ages zero to 5 years, and the median age at female genital mutilation retrieved from the women’s module is sensitive to this assumption. However, the difference between data for women and daughters is in line with previous research in Senegal^43^ and neighbouring countries such as Gambia.^44^

Previous research shows that changes in the age at female genital mutilation over time are rooted in context-specific evolving social norms and realities.^44^ Studies in the Kolda region in Senegal found declining age at female genital mutilation.^45^ UNICEF also found declining ages in Benin, Burkina Faso, Central African Republic, Côte d’Ivoire, Gambia, Guinea, Guinea-Bissau, Kenya, Mali, Nigeria and Sierra Leone, but no statistically significant change for other countries such as Senegal.^19^ In contrast, DHS data from Egypt demonstrated increasing age at experiencing female genital mutilation.^18^

Our analysis demonstrates that the changes observed in age at female genital mutilation in our data set are sensitive to how the data were obtained, that is, either proxy-reported or self-reported. With more data on women in older age cohorts and more data on daughters in younger cohorts, this factor may bias trend estimates for age at female genital mutilation. This finding highlights that the women’s and daughters’ modules cannot be easily compared without taking the differences in their data quality into account.

Our study has some limitations. First, while data on age at female genital mutilation were missing in some records, and this amount was greater than for other variables in the data set (e.g. educational attainment and birth cohort), the overall level of complete missing seems moderate. However, 35–60% of women provided an approximate answer to their age at experiencing female genital mutilation, namely during infancy (online repository).^28^ To include these observations in our analysis, we had to make assumptions on how to interpret infancy and opted for the wide age range of 0–5 years. However, we did not have evidence on how survey respondents interpreted infancy. Future research might be able to establish exactly how to convert approximate answers with respect to age at female genital mutilation into appropriate age ranges.

Second, female genital mutilation is context specific. Our analysis focused on female genital mutilation data in Senegal and its findings may be restricted to this context. In Senegal, female genital mutilation occurs mostly in early childhood. Even though the age of women was not a relevant predictor of the accuracy of reporting age at experiencing female genital mutilation in our analysis, the difference between women and daughters may be more relevant in a context with young age at female genital mutilation compared to one where female genital mutilation occurs later in life. For example, a decrease in the age at female genital mutilation in Kenya within the women’s module was found in a previous study, indicating a decline in the age independent of survey module.^21^

To conclude, our analysis suggests caution against only using the women’s module (i.e. self-reported data on female genital mutilation) to assess age at occurrence of the practice, at least when female genital mutilation occurs early in life. While we expected lower data quality for self-reported data by women on themselves, our analysis shows how important this problem can be when assessing the age pattern of female genital mutilation and how it may distort conclusions about the level and temporal trend of age at female genital mutilation based on survey data.

Our findings are relevant to both research and the design of programmes to end harmful practices such as female genital mutilation. We recommend an analysis of age at female genital mutilation including trends over time by survey module and, where data are available, primarily based on the daughters’ module. This analysis is possible for Senegal as data on female genital mutilation from both modules are available and the age for daughters covers the birth cohorts from the 1990 to the 2020s. Such analyses should closely evaluate the quality of reporting of age at female genital mutilation for women and daughters and adjust the interpretation of the data accordingly.

## References

[1] Female genital mutilation. Geneva: World Health Organization; 2023. Available from: https://www.who.int/news-room/fact-sheets/detail/female-genital-mutilation [cited 2023 Oct 8].

[2] Weny K, Silva R, Snow R, Legesse B, Diop N. Towards the elimination of FGM by 2030: a statistical assessment. PLoS One. 2020 Oct 6;15(10):e0238782. 10.1371/journal.pone.0238782 33021973 PMC7537854

[3] Weny K, Silva R, Diop N, Snow R. Spatial clustering in temporal trends of female genital mutilation risk: leveraging sparse data in Ethiopia, Kenya, and Somalia. Stud Fam Plann. 2023 Sep;54(3):487–501. 10.1111/sifp.12242 37370236

[4] Eliminating female genital mutilation – an interagency statement. Geneva: World Health Organization; 2008. Available from: https://www.who.int/fr/publications/i/item/9789241596442 [Cited 2024 Jun 16].

[5] UNFPA-UNICEF joint programme on the elimination of female genital mutilation. New York: United Nations Population Fund; 2023. Available from: https://www.unfpa.org/unfpa-unicef-joint-programme-female-genital-mutilation-1 [cited 2023 Oct 13].

[6] Resolution A/RES/70/1. Transforming our world: the 2030 agenda for sustainable development. In: Seventieth United Nations General Assembly, New York, 25 September 2015. New York: United Nations; 2015. Available from: https://www.un.org/en/development/desa/population/migration/generalassembly/docs/globalcompact/A_RES_70_1_E.pdf [cited 2023 August 20].

[7] Female genital mutilation (FGM) data. New York: United Nations Children’s Fund; 2023. Available from: https://data.unicef.org/topic/child-protection/female-genital-mutilation/ [cited 2023 Oct 12].

[8] FGM prevalence among girls and women aged 15 to 49 years, by residence and wealth quintile (%). New York: United Nations Children’s Fund; 2023. Available from: https://data.unicef.org/topic/child-protection/female-genital-mutilation/ [cited 2024 Feb 29].

[9] FGM prevalence among girls aged 0 to 14 years, by residence and wealth quintile (%). New York: United Nations Children’s Fund; 2023. Available from: https://data.unicef.org/topic/child-protection/female-genital-mutilation/ [cited 2024 Feb 29].

[10] De Schrijver L, Van Baelen L, Van Eekert N, Leye E. Towards a better estimation of prevalence of female genital mutilation in the European Union: a situation analysis. Reprod Health. 2020 Jul 8;17(1):105. 10.1186/s12978-020-00947-2 32641062 PMC7341583

[11] Besera G, Goldberg H, Okoroh EM, Snead MC, Johnson-Agbakwu CE, Goodwin MM. Attitudes and experiences surrounding female genital mutilation/cutting in the United States: a scoping review. J Immigr Minor Health. 2023 Apr;25(2):449–82. 10.1007/s10903-022-01437-2 36542264 PMC10981529

[12] Uganda demographic and health survey 2016. Kampala and Rockville: Uganda Bureau of Statistics (UBOS) and ICF International; 2018.

[13] Northeast Zone multiple indicator cluster survey 2011, final report. Nairobi: United Nations Children’s Fund, Somalia, and Ministry of Planning and International Cooperation; 2014.

[14] Somaliland multiple indicator cluster survey 2011, final report. Nairobi: United Nations Children’s Fund, Somalia, and Somaliland Ministry of Planning and National Development; 2014.

[15] Sénégal: enquête démographique et de santé continue (EDS-continue). Dakar and Rockville: Agence Nationale de la Statistique et de la Démographie (ANSD) and ICF International; 2023.

[16] Female genital mutilation in Senegal. Insights from a statistical analysis. New York: United Nations Children’s Fund; 2022. Available from: https://data.unicef.org/resources/female-genital-mutilation-in-senegal/ [cited Feb 16 2025].

[17] Tailoring steps to end female genital mutilation based on age. New York: United Nations Population Fund; 2020. Available from: https://www.unfpa.org/resources/tailoring-steps-end-female-genital-mutilation-based-age [cited 2023 Aug 10].

[18] Yoder SP, Wang S. Female genital cutting: the interpretation of recent DHS data. DHS Comparative Reports No. 33. Calverton: ICF International; 2013.

[19] The power of education to end female genital mutilation. New York: United Nations Children’s Fund; 2022. Available from: https://data.unicef.org/resources/the-power-of-education-to-end-female-genital-mutilation/ [cited 2023 Aug 20].

[20] Yoder SP, Abderrahim N, Zhuzhuni A. Female genital cutting in the demographic and health surveys: a critical and comparative analysis. DHS comparative reports No 7. Calverton: ORC Macro; 2004.

[21] Shell-Duncan B, Naik R, Feldman-Jacobs C. A state-of-the-art synthesis on female genital mutilation/cutting: what do we know now? New York: Population Council; 2016. 10.31899/rh8.1002

[22] Shell-Duncan B. Considerations on the use and interpretation of survey data on FGM/C. Technical brief, evidence to end FGM/C: research to help women thrive. New York: Population Council; 2016. 10.31899/rh8.1003

[23] Measuring effectiveness of female genital mutilation elimination: a compendium of indicators. UNFPA-UNICEF joint programme on the elimination of female genital mutilation: accelerating change. New York: United Nations Population Fund; 2020. Available from: https://www.unfpa.org/sites/default/files/pub-pdf/026_UF_CompendiumOfIndicatorsFGM_21-online_F.pdf [cited 23 Feb 2025].

[24] The DHS program Senegal. Washington, DC: USAID; 2023. Available from: https://dhsprogram.com/Countries/Country-Main.cfm?ctry_id=36&c=Senegal [cited 2023 Oct 13].

[25] Ndiaye S, Ayad M. Enquête démographique et de santé au Sénégal 2005. Dakar and Calverton: Centre de Recherche pour le Développement Humain and ORC Macro; 2006.

[26] Sénégal: enquête démographique et de santé continue (EDS-continue) 2012–2013. Dakar and Calverton: Agence Nationale de la Statistique et de la Démographie (ANSD) and ICF International; 2012.

[27] Engelsma B, Mackie G, Merrell B. Unprogrammed abandonment of female genital mutilation/cutting. World Dev. 2020;129:104845. 10.1016/j.worlddev.2019.104845

[28] Weny K, Silva R, Klug SJ. Self-report and proxy reports in survey data on female genital mutilation, Senegal. Supplementary material [online repository]. Charlottesville, VA: Center for Open Science; 2025. 10.17605/OSF.IO/ETW9F PMC12161157 40511400

[29] Pullum TW. An assessment of age and date reporting in the DHS surveys, 1985–2003. Methodological reports no. 5. Calverton: Macro International Inc.; 2006.

[30] Lumley T, Gao P, Schneider B. Analysis of complex survey samples. Package ‘survey’ [internet]. Vienna: The Comprehensive R Archive Network; 2024. Available from: https://cran.r-project.org/web/packages/survey/survey.pdf [cited 2024 Aug 20].

[31] Sénégal: enquête démographique et de santé à indicateurs multiples au Sénégal (EDS-MICS) 2010–2011. Dakar and Calverton: Agence Nationale de la Statistique et de la Démographie and ICF International; 2012.

[32] Sénégal: enquête démographique et de santé continue (EDS-continue) 2014. Dakar and Rockville: Agence Nationale de la Statistique et de la Démographie and ICF International; 2015.

[33] Sénégal: enquête démographique et de santé continue (EDS-continue) 2015. Dakar and Rockville: Agence Nationale de la Statistique et de la Démographie and ICF International; 2016.

[34] Sénégal: enquête démographique et de santé continue (EDS-continue) 2016. Dakar and Rockville: Agence Nationale de la Statistique et de la Démographie and ICF International; 2017.

[35] Sénégal: enquête démographique et de santé continue (EDS-continue) 2017. Rockville: Agence Nationale de la Statistique et de la Démographie and ICF; 2018.

[36] Sénégal: enquête démographique et de santé continue (EDS-continue) 2018. Rockville: Agence Nationale de la Statistique et de la Démographie and ICF; 2018.

[37] Sénégal: enquête démographique et de santé continue (EDS-continue) 2019. Rockville: Agence Nationale de la Statistique et de la Démographie and ICF; 2019.

[38] Female genital mutilation/cutting – a statistical overview and exploration of the dynamics of change. New York: United Nations Children’s Fund; 2013. Available from: https://data.unicef.org/resources/fgm-statistical-overview-and-dynamics-of-change/ [cited 2023 Aug 10].

[39] Klouman E, Manongi R, Klepp KI. Self-reported and observed female genital cutting in rural Tanzania: associated demographic factors, HIV and sexually transmitted infections. Trop Med Int Health. 2005 Jan;10(1):105–15. 10.1111/j.1365-3156.2004.01350.x 15655020

[40] Bjälkander O, Grant DS, Berggren V, Bathija H, Almroth L. Female genital mutilation in Sierra Leone: forms, reliability of reported status, and accuracy of related demographic and health survey questions. Obstet Gynecol Int. 2013;2013:680926. 10.1155/2013/680926 24204384 PMC3800578

[41] Basannar DR, Singh S, Yadav J, Yadav AK. Quantifying age heaping and age misreporting in a multicentric survey. Indian J Community Med. 2022 Jan–Mar;47(1):104–6. 10.4103/ijcm.ijcm_1179_21 35368490 PMC8971874

[42] Fayehun O, Ajayi AI, Onuegbu C, Egerson D. Age heaping among adults in Nigeria: evidence from the Nigeria demographic and health surveys 2003–2013. J Biosoc Sci. 2020 Jan;52(1):132–9. 10.1017/S0021932019000348 31339087

[43] Matanda D, Atilola G, Moore Z, Komba P, Mavatikua L, Nnanatu CC, et al. Female genital mutilation/cutting in Senegal: is the practice declining? Descriptive analysis of demographic and health surveys, 2005–2017. Evidence to End FGM/C: research to help girls and women thrive. New York: Population Council; 2020.

[44] Shell-Duncan B, Wander K, Hernlund Y, Moreau A. Dynamics of change in the practice of female genital cutting in Senegambia: testing predictions of social convention theory. Soc Sci Med. 2011 Oct;73(8):1275–83. 10.1016/j.socscimed.2011.07.022 21920652 PMC3962676

[45] Camilotti G. Interventions to stop female genital cutting and the evolution of the custom: evidence on age at cutting in Senegal. J Afr Econ. 2016;25(1):133–58. 10.1093/jae/ejv013

